# Distinct antibody response in susceptible and non-susceptible hosts of the carcinogenic liver fluke *Opisthorchis viverrini* infection

**DOI:** 10.1017/S0031182023000112

**Published:** 2023-07

**Authors:** Krongkarn Watakulsin, Arpa Surapaitoon, Lorina Handayani Ulag, Sunheng Kaing, Watcharapol Suyapoh, Prasert Saichua, Kanin Salao, Sirikachorn Tangkawatana, Sutas Suttiprapa

**Affiliations:** 1 Department of Tropical Medicine, Faculty of Medicine, Khon Kaen University, Khon Kaen 40002, Thailand; 2 Tropical Disease Research Center, WHO Collaborating Centre for Research and Control of Opisthorchiasis, Faculty of Medicine, Khon Kaen University, Khon Kaen 40002, Thailand; 3 Department of Microbiology, Faculty of Medicine, Khon Kaen University, Khon Kaen 40002, Thailand; 4 Department of Biomedicine, School of Life Sciences, Indonesia International Institute for Life Sciences, Jakarta 13210, Indonesia; 5 Faculty of Veterinary Science, Prince of Songkla University, Songkhla 90110, Thailand; 6 Faculty of Veterinary Medicine, Khon Kaen University, Khon Kaen 40002, Thailand

**Keywords:** Antibody, carcinogenic, liver fluke, non-susceptible, *Opisthorchis viverrini*, susceptible

## Abstract

*Opisthorchis viverrini* is a carcinogenic parasite that can cause bile duct cancer called cholangiocarcinoma. A study of the immune response of this parasite in susceptible and non-susceptible hosts may provide a clue to develop vaccines and immunodiagnostic markers, which are currently not available. Here, we compared the antibody response in susceptible Golden Syrian hamsters and non-susceptible BALB/c mice infected by the liver fluke. In mice, the antibody was detected between 1 and 2 weeks post-infection, whereas it was positive between 2 and 4 weeks post-infection in hamsters. Immunolocalization revealed that the antibody from mice reacts strongly with the tegumental surface and gut epithelium of the worm, while hamster antibody showed a weak signal in the tegument and a comparable signal in the gut of the worm. Immunoblot of the tegumental proteins demonstrated that while hamster antibody showed a broad specificity, mice strongly reacted with a single protein band. Mass spectrometry revealed these immunogenic targets. Recombinant proteins of the reactive targets were produced in the bacterial expression system. The immunoblot of these recombinant proteins confirm the reactivity of their native form. In summary, there is a different antibody response against *O. viverrini* infection in susceptible and non-susceptible hosts. The non-susceptible host reacts quicker and stronger than the susceptible host.

## Graphical abstract

**Figure:**
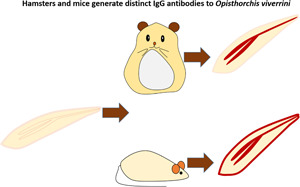

## Introduction

*Opisthorchis viverrini* is a human liver fluke that causes serious public health concerns in some parts of Southeast Asia, including Thailand, Laos PDR, Vietnam and Cambodia (Sripa *et al*., 2021). The *O. viverrini* infection is prevalent in regions where the consumption of raw freshwater fish is commonly found. The infection of *O. viverrini* mainly causes opisthorchiasis associated with several hepatobiliary diseases including cholangitis, obstructive jaundice, hepatomegaly, cholecystitis and cholelithiasis. Moreover, *O. viverrini* infection is strongly associated with bile duct cancer, cholangiocarcinoma which is unprecedentedly high in these regions (Sripa *et al*., 2018).

Despite genome, transcriptome and proteome data of this parasite are now publicly available (Mulvenna *et al*., 2010*b*; Young *et al*., 2010, 2014). However, there is still no effective immunodiagnostic test for *O. viverrini* infection. In addition, there is no vaccine against this carcinogenic parasite. This is partly because we do not fully understand the immune response of this worm. Studies in other parasitic infections demonstrated that comparing the antibody response between susceptible and non-susceptible/resistant hosts may yield immunogenic molecules that can be developed as vaccine targets and diagnostic markers (Pearson *et al*., 2015; Driguez *et al*., 2016).

Therefore, we aimed to compare the antibody response between Golden Syrian hamsters which is a susceptible host, and BALB/c mice which are non-susceptible hosts by employing several approaches, including enzyme-linked immunosorbent assay (ELISA), western blot, immunohistochemistry (IHC) and mass spectrometry.

## Materials and methods

### Ethics approval, animal infection and specimen collection

The study protocol was approved by the Khon Kaen University Animal Ethics Committee for the project titled ‘Comparative Immunopathology of Liver Fluke Infection in Susceptible and Non**-**Susceptible Animal Models’ (AEKKU 78**/**61, IACUC KKU 57**/**2563). Golden Syrian hamsters (*Mesocricetus auratus*) were maintained at the animal research facility of the Khon Kaen University, Faculty of Medicine. BALB/c mice were housed at the Northeast Laboratory Animal Center, Khon Kaen University. A group of 5 male hamsters and 5 male mice per time point were infected with 50 metacercariae of *O. viverrini* by intragastric intubation according to the standard protocol (Lvova *et al*., 2012) and anaesthetized with isoflurane and sacrificed at day 1, day 2, day 7, day 14, day 28 and day 56 post-infection for collection of blood *via* cardiac puncture, adult worms and tissue samples. Another group of 5 animals per time point without *O. viverrini* infection was used as the control group. Blood samples were allowed to clot by incubating at room temperature (RT) for 1 h. The clot was removed by centrifugation at 1000–2000 ***g*** for 10 min in a refrigerated centrifuge. The supernatant derived from sera was separated into aliquots and labelled with a unique individual identification code and maintained at −20°C until further use. Adult worms and tissues were fixed with 10% neutral-buffered formalin overnight at RT and embedded in paraffin blocks. The tissue blocks were cut into 4-*μ*m thickness and stained with haematoxylin and eosin for histologic screening.

### Preparation of parasite antigen

Crude somatic *O. viverrini* (SomOV) antigen was prepared from adult worms obtained from experimentally infected hamsters at 4-week and 8-week post-infection. Adult worms in phosphate-buffered saline (PBS) pH 7.4 containing protease inhibitors [0.1 mm phenyl methyl sulphonyl fluoride, 0.1 mm *trans*-epoxysuccinyl-l-leucylamido(4-guanidino)butane] were homogenized and sonicated (Sonics & Materials Inc., CT, USA). The sonicated material was then centrifuged at 15 000 rpm at 4°C for 30 min. The soluble protein in the supernatant is considered as crude somatic *O. viverrini* antigen, and the antigen was stored at −70°C for subsequent use.

### ELISA of somatic antigens

Specific antibody against SomOV was measured in the serum by indirect ELISA. Maxisorp flat-bottom 96-well microtitre plates (MaxiSorp; Nunc, Roskilde, Denmark) were coated with 1 *μ*g of crude SomOV antigen mL^−1^ (100 *μ*L well^−1^) in coating buffer (50 mm carbonate buffer, pH 9.6) and incubated overnight at 4°C. Plates were washed with PBS containing 0.05% Tween-20 (PBST) and then blocked with 5% skim milk in carbonate buffer, pH 9.6 for 1 h at 37°C. One hundred microlitres of diluted sera (1:50 in PBST/2% skim milk) from infected and non-infected mice and hamsters were added and incubated for 1 h at 37°C. The plates were washed with PBST and probed with 100 *μ*L of diluted polyclonal goat anti-mouse immunoglobulin G (IgG) (H + L)–horseradish peroxidase (HRP) (Thermo Fisher Scientific Inc., catalogue no. 31431), or polyclonal goat anti-hamster IgG (H + L)–HRP (Thermo Fisher Scientific, catalogue no. PA1-28823) for 1 h at 37°C. After washing with PBST, the colour was developed by adding 100 *μ*L of 3,3′,5,5′-tetramethyl benzidine (TMB) (BioLegend, USA) for 20 min at RT, and reaction was stopped by adding 50 *μ*L of 0.5 m H_2_SO_4_. The colorimetric reaction was read at a wavelength of 450 nm on a VersaMax Microplate Reader (Molecular Devices, USA). Each sample was assayed in duplicate.

### Western blot analysis of somatic antigens

SomOV antigens were electrophoresed on sodium dodecyl sulphate-polyacrylamide gel electrophoresis (SDS-PAGE) and transferred to nitrocellulose western blotting membranes (Amersham, UK). The membranes were cut into strips and blocked overnight at 4°C in 5% skimmed milk/PBST. Following a gentle rinse with PBST at RT, the strips were incubated for 1 h at RT with rabbit anti-SomOV antibody (a gift from Professor Sripa) (Sripa and Kaewkes, 2000*a*) (1:1000) or diluted sera (1:100) from 4-weeks *O. viverrini* infected and non-infected mice and hamster dilutions in 2% skimmed milk/PBST. The strips were further incubated with the following secondary antibodies, polyclonal goat anti-rabbit IgG (H + L)–HRP (Abcam, catalogue no. ab6721), polyclonal goat anti-mouse IgG (H + L)–HRP (Thermo Fisher Scientific Inc., catalogue no. 31431), or polyclonal goat anti-hamster IgG (H + L)–HRP (Thermo Fisher Scientific, catalogue no. PA1-28823) as a secondary antibody (1:1000 dilutions) for 1 h at RT. After each incubation step, the strips were washed thrice. Finally, the reactive protein bands were visualized with a commercial chemiluminescent substrate (SuperSignal™ West Pico PLUS Chemiluminescent Substrate, Pierce, USA), and the photographs were captured using an AI600 Chemiluminescent Imager (Amersham, USA). The reactions without primary antibodies (mouse or hamster sera) were tested to ensure that the difference was not derived from the detection antibodies. Another SDS-PAGE gel of somatic antigens was stained with 0.1% Coomassie blue R250 in 10% acetic acid, 50% methanol for half an hour and then destained with 10% acetic acid, 50% methanol until the background is nearly clear.

### Immunohistochemical analysis

The paraffin sections of *O. viverrini* were deparaffinized with xylene and then hydrated in a series of ethanol and distilled water. The antigen was retrieved by incubating sections with citrate buffer (pH 6.0) at 121°C for 5 min, and then washed in PBS. Endogenous peroxidase was eliminated by treating tissue sections with absolute methanol containing 5% H_2_O_2_ for 30 min. Non-specific staining was blocked by treating slides with 5% normal horse serum in NaN_3_/PBS for 30 min. Subsequently, the sections were probed with anti-SomOV antibody (1:500 in NaN_3_/PBS) and diluted sera (1:40 in NaN_3_/PBS) of 4-weeks post-infected, non-infected mice and 4-weeks post-infected, non-infected hamsters and incubated at RT overnight in a moist chamber. The diluted-goat anti-rabbit IgG–HRP, goat anti-hamster IgG–HRP and goat anti-mouse IgG–HRP (1:300) were added onto sections and incubated at RT for 1 h in a moist chamber. After that, the tissue sections were rinsed with PBS, and the colour was developed by adding 0.05% diaminobenzidine tetrahydrochloride in 0.003% H_2_O_2_ solution for 2 min. The slides were counterstained with Mayer's haematoxylin, dehydrated, cleared in xylene and mounted with Permount^®^ (Fisher Scientific, USA). The sections were observed, and images were captured with an imager microscope (Axio Imager, Zeiss, Germany). This descriptive study was performed under the semi-quantitative evaluation. Brown stain indicated a positive reaction whereas no brown stain indicated a negative reaction. The IHC interpretation was made by 2 veterinary pathologists with blinded scoring. The histological examination of IHC staining was performed at 2 consecutive sections with 5–10 locations. The semi-quantitative scoring system was based on Upontain *et al*. (2018). Briefly, the staining intensity was recorded as 0 = no staining, 1 = mild, 2 = moderate, 3 = strong, under a light microscope. The data were statistically analysed using SPSS version 23.0 (SPSS Inc., USA). The analysis of variance was used to compare various groups. The *P* values <0.05 were considered as statistically significant.

### *Opisthorchis viverrini* tegumental protein extraction

One hundred and fifty *O. viverrini* adult worms were used for tegumental protein extraction. The teguments of worms were removed by freeze–thawing technique (Mulvenna *et al*., 2010*b*). Briefly, worms were soaked in PBS with protease inhibitor (E-64). After that, they were frozen in liquid nitrogen and thawed on ice thrice, followed by a short burst with a vortex to release the tegument. The buffer containing the tegument was collected and centrifuged at 12 000 rpm at 4°C for 30 min to obtain the tegumental pellet. The pellet was resuspended in 40 mm Tris, pH 7.4, and incubated on ice for 20 min. Then it was centrifuged at 15 000 rpm for 20 min at 4°C and the supernatant was collected. The procedure was repeated twice with 40 mm Tris, pH 7.4, and the 3 supernatants were pooled. The pellet was then solubilized with 5 m urea in 40 mm Tris, pH 7.4 and 0.1% SDS, 1% Triton X-100 in 40 mm Tris, pH 7.4, with the same protocol as for 40 mm Tris, pH 7.4. Three supernatants from each buffer were collected and pooled. All the supernatants were stored at −80°C until further use.

### SDS-PAGE and western analysis of tegumental proteins

The supernatants from 3 extracts (40 mm Tris, 5 m in 40 mm Tris urea and 0.1% SDS, 1% Triton X-100 in 40 mm Tris) were separated by SDS-PAGE. The supernatants were mixed with 4× Laemmli sample buffer (250 mm Tris, pH 6.8, 8% SDS, 0.1% bromophenol blue, 20% *β*-mercaptoethanol) and resolved by 9% SDS-PAGE. The separated proteins were transferred onto the polyvinylidene difluoride membrane (Immobilon-P) and then blocked in 5% bovine serum albumin (BSA) in 1× PBS, incubated overnight at 4°C. The membrane was incubated in pooled 4-week *O. viverrini*-infected mouse serum (1:250) and in pooled 4-week *O. viverrini*-infected hamster serum (1:250) in 2% BSA containing 0.05% Tween-20 in 1× PBS, and gently shaken for 2 h at RT. The membrane was washed thrice in 0.05% Tween-20 in 1× PBS and then incubated in goat anti-mouse IgG (H + L) peroxidase-conjugated or goat anti-hamster IgG (H + L) (1:1000), HRP conjugated (1:1000) in 1× PBS for 1 h at RT. At the end of incubation, the membrane was washed twice and equilibrated in 1× PBS until detection. The colorimetric signal was detected using Pierce™ ECL Plus Western Blotting Substrate (Thermo Fisher, USA). The protein bands on SDS-PAGE corresponding to the reactive bands on the western blot were excised and subjected to mass spectrometry for protein identification.

### Mass spectrometry by LC-ESI-MS/MS analysis

Samples were analysed with a nano-liquid chromatography system (EASY-nLC II, Bruker, MA, USA) coupled to an ion trap mass spectrometer (Amazon Speed ETD, Bruker) equipped with an ESI nano-sprayer (ESI-TRAP). The ESI-TRAP instrument was calibrated in the *m*/*z* range 50–3000 using an internal calibration standard (tune mix solution) which is supplied from Agilent. A sample volume of 10 *μ*L was loaded by the autosampler onto an EASY-Column, 10 cm, ID 75 *μ*m, 3 *μ*m, C18-A2 (Thermo Scientific) at a flow rate of 500 nL min^−1^. Mobile phase A was water with 0.1% formic acid and B was acetonitrile with 0.1% formic acid. The gradient was 5–35% B in 50 min followed by 10 min at 80% B. The 100 fmol tryptic digest of BSA used as a standard control for the experiment. Bruker Daltonics software package HyStar v.3.2 was used to control the Qq-TOF device. LC-MS/MS spectra were analysed using Compass Data Analysis v.4.0. Compound lists were exported as Mascot generic files (mgf) for further searching in MASCOT program.

### Data analysis

Protein identification was performed by searching against different protein databases from NCBIprot or SwissProt (other metazoa) using an in-house MASCOT server (Matrixscience, London, UK) and MASCOT MS/MS Ion Search program (www.matrixscience.com) with the initial searching parameters: enzyme: trypsin; carbamidomethylation (C) as fixed modification and oxidation (HW) and oxidation (M) as variable modification; peptide mass tolerance of 0.5 Da and fragment mass tolerance of 0.5 Da; a peptide charge state of +1, +2, +3; instrument type: ESI-TRAP and report top: Auto.

### Recombinant protein production of tegumental proteins

The nucleotide sequence encoding the identified putative tegumental proteins [M60-1, succinate dehydrogenase (SDH), calpain, adenosine triphosphate (ATP) synthase and innexin] was synthesized and sub-cloning into pET21a(+) by U2Bio Co., Ltd, Korea. Recombinant plasmids were used to transform BL21(DE3) chemically competent cells. Protein expressions were induced by 0.5 mm isopropyl *β*-d-1-thiogalactopyranoside at 37°C, 200 rpm overnight. Culture media were centrifuged at 4000 rpm, 4°C for 10 min (Allegra R15XSX2750, Beckman Coulter Inc., USA) to collect bacterial pellets. The pellet was resuspended in lysis buffer containing 0.5 m NaCl, 20 mm Tris-HCl (pH = 7.5), 1% SDS, and 8 m urea, and sonicated at 4°C for 5 min (1 s ON, 5 s OFF). The cell lysate was centrifuged at 12 000 rpm at 4°C for 30 min to collect the supernatant containing solubilized protein. The proteins were separated by SDS-PAGE (5% stacking gel, 12.5% separating gel). The gel was incubated with 0.1 m KCl at 4°C for 1 h. Recombinant protein bands at expected size were excised from the gel and homogenized in 500 *μ*L of lysis buffer. Then it was incubated at RT overnight with gentle shaking and spin down at 14 000 rpm for 5 min to collect the supernatant containing partially purified recombinant protein. The proteins were buffer-exchanged into PBS and stored at −80°C until further use.

### ELISA of the recombinant tegumental proteins

The 96-well plates were coated with 1 mg well^−1^ 100 *μ*L^−1^ recombinant proteins (M60-1, SDH, calpain, ATP synthase and innexin) in coating buffer (0.05 m carbonate buffer, pH 9.6) and incubated overnight at 4°C. The coated plates were washed thrice with PBST and blocked with 200 *μ*L well^−1^ of 5% BSA in 1× PBS and incubated for 2 h at RT. Blocking buffer was replaced by 100 *μ*L primary antibody (pooled non-infected and infected hamster and mouse at week 1, 2 and 4) diluted 1:100 in 10% fetal bovine serum in 1× PBS and incubated at RT for 1 h. After washing with PBST thrice, the plates were incubated with HRP-conjugated goat anti-hamster IgG (H + L) (Invitrogen, USA) at 1:1000 dilution or HRP-conjugated goat anti-mouse IgG (H + L) (Invitrogen, USA) at 1:1000 dilution for 1 h at RT. At the end of incubation, plates were washed with PBST, and TMB substrate (BioLegend, USA) was added. The plates were incubated at RT for 15 min in the dark, and the reactions were stopped by adding 2 N H_2_SO_4_. The colorimetric reaction was read at 450 nm by using a VersaMax Microplate Reader (Molecular Devices, USA). Each sample was assayed in duplicate.

## Results

### Antibody responses were delayed in *O. viverrini*-infected hamsters

**Figure 1.**
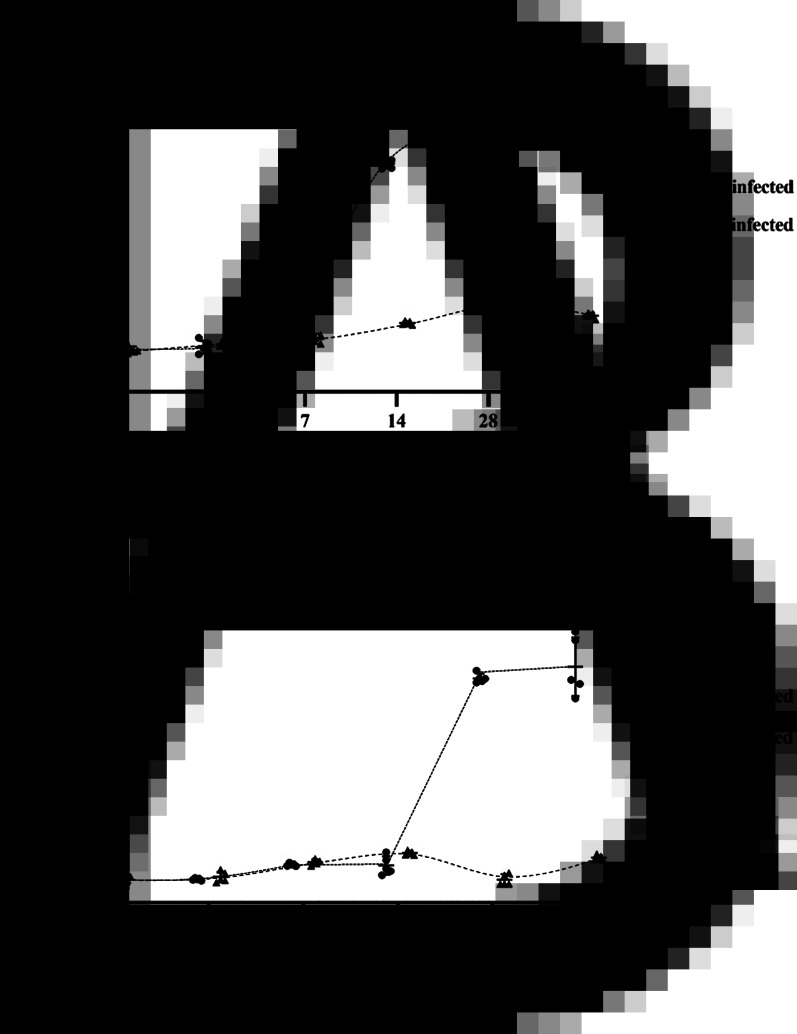
Serum antibody response to somatic antigens in hamsters and mice non-infected and infected by *Opisthorchis viverrini* at day 1, day 2, day 7, day 14, day 28 and day 56 post-infection. Results represent the mean ± s.d. of absorbance measured at 450 nm for each group (5 animals per group).

The whole worm somatic extracts were used for detection of antibody response in *O. viverrini* infection. Figure 1 shows the antibodies from mice and hamster infected and non-infected by *O. viverrini*. ELISA results show that the mean of OD levels of antibodies against the *O. viverrini* somatic extracts was higher in the mice that were infected than the uninfected mice. The mean of OD levels of antibodies in mice increased at 2 weeks after infection and continued until week 8 of *O. viverrini* infection. Similar to mice, the antibodies against *O. viverrini* were higher in the hamsters that were infected than the uninfected mice. In contrast, the antibodies in the hamsters were not detected at week 2 of infection. However, it increased sharply at 4 weeks after infection. There was a slight increase in the antibody at week 8 of infection in both mice and hamsters.

### Hamsters and mice reacted with high molecular weight proteins of somatic extracts

**Figure 2.**
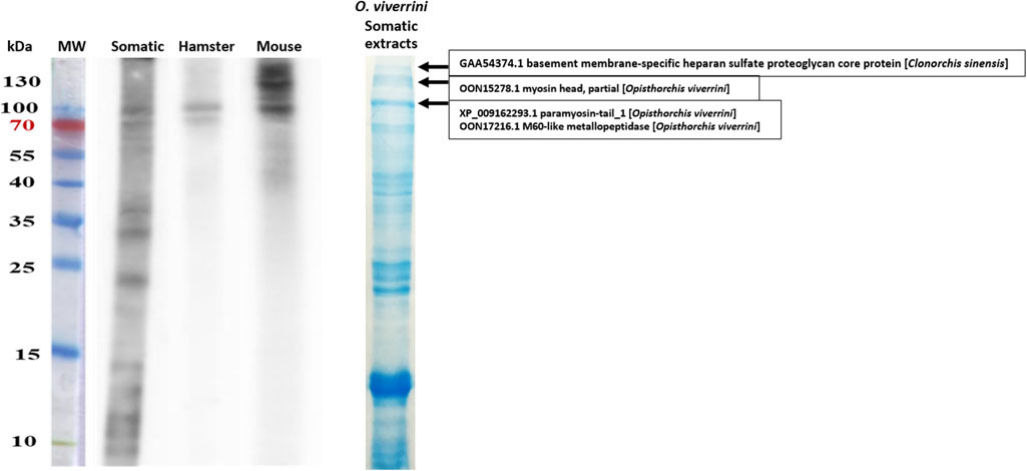
(Left) Western blot analysis of *O. viverrini* somatic extracts to determine the reactive proteins recognize by rabbit anti-somatic extracts IgG (somatic), 4-week *O. viverrini*-infected sera of hamsters (hamster) and mice (mouse). (Right) SDS-PAGE gel of somatic extract stained by Coomassie blue, and protein identified by mass spectrometry at different sizes.

To determine proteins that were recognized by the antibody, we performed a western blot analysis. The protein fractions ranging from 10 to 130 kDa were separated from the somatic extracts of *O. viverrini* by SDS-PAGE (Fig. 2). The western blot result shows different patterns of positive reactivity among sera. The rabbit anti-somatic extracts which were used as a positive control reacted with the proteins at various molecular weights ranging from 10 to 100 kDa. Contrarily, sera from infected hamsters reacted with two protein bands at molecular weights between 70 and 100 kDa. Intriguingly, mouse sera reacted strongly with protein fractions at 100 kDa and above. To identify these fractions, we performed mass spectrometry and found that the fraction at 100 kDa was paramyosin tail_1 (XP_009262293.1) and M60-like metallopeptidase (OON17216.1). In addition, the protein at 130 kDa was myosin head (OON15278.1), and the band above 130 kDa was a basement-specific heparan sulphate proteoglycan core protein (GAA54374.1).

### Antibody from mice reacted strongly with the tegument while hamster antibody preferentially bound to the gut of the worm

**Figure 3.**
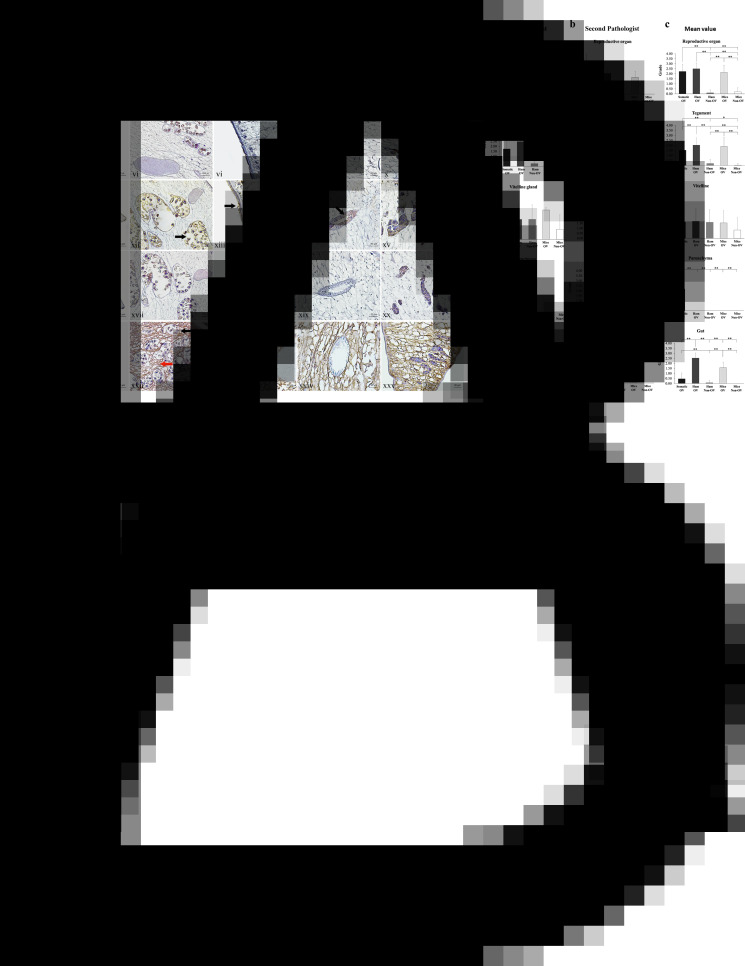
(A) Immunoperoxidase localization on paraffin embedded *O. viverrini* tissue. Panels i–v, *O. viverrini*-infected hamster serum; vi–x, non-infected hamster serum; xi–xv, *O. viverrini*-infected mice serum; xvi–xx, non-infected mice serum; xxi–xxv, rabbit anti-somatic extracts of *O. viverrini*. Reproductive organs were strongly positive in the uteri and eggs (black arrow) of the *O. viverrini*-infected hamster and mice sera (ii, xii), and the rabbit anti-somatic extracts of *O. viverrini* (xxii). The positive signals of tegument (black arrow) and tegumental cells (red arrow) were detected in all *O. viverrini*-infected serum (iii, xiii, xxiii). The strongest positive signal illustrated in *O. viverrini*-infected mice serum (xiii). Gut of the worm show positive signal in the *O. viverrini*-infected hamster and mice sera (black arrow; iv, xiv). Vitelline glands show non-specific binding in all serum-treated groups (v, x, xv, vx, xxv). Only rabbit anti-somatic extracts of *O. viverrini* show strongly positive in parenchyma (red arrow; xxii). The original magnifications: iii–v, vi–x, xiii–xv, xviii–xx, xxiii–xxv = ×40, scale bar = 20 *μ*m; ii, vi, xii, xvii, xxii = ×10, scale bar = 100 *μ*m; i, vi, xi, xvi, xxi = ×5, scale bar = 200 *μ*m. (B) Scoring the immunohistochemical intensity by 2 independent assessors: (a) scoring by the first pathologist; (b) scoring by the second pathologist and (c) mean values from pathologists with the semi-quantitative comparison of colour intensities. Somatic OV, anti-SomOV antigen; Ham OV, diluted sera of infected hamster; Ham Non-OV, diluted sera of non-infected hamsters; Mice OV, diluted sera of infected mice; Mice Non-OV, diluted sera of non-infected mice (*P < 0.05; **P < 0.01).

Figure 3 demonstrates the staining pattern of non-infected and infected sera from hamsters and mice on the tissue of *O. viverrini* by immunoperoxidase. Five different areas that were assessed include worm parenchyma, reproductive system (ovary and seminal receptacle), tegument and tegumental cells, gut epithelium and vitelline glands. Overall, *O. viverrini*-infected sera show multi-area positive, while no obvious staining was detected by non-infected sera. Hamster sera stained most of the areas except parenchymal tissue (Fig. 3i–v). The strong staining was seen in the gut and secretion in the uteri and inside the eggs. In the gut, the staining was detected in both epithelial cytoplasm and secretion in the gut lumen (Fig. 3iv). There was a scattered staining pattern on the tegument surface and some in the tegumental cell bodies (Fig. 3iii); however, the intensity was not as strong as in the gut. The staining pattern of *O. viverrini*-infected mice sera was similar to that of the hamster. However, the main difference was the strong signal on both tegumental surface and tegumental cells. In the gut, the staining was limited only to the epithelium cytoplasm, no positive reaction in the gut lumen as seen in the hamster serum staining. Vitelline glands show non-specific binding as all sera show positive immunoperoxidase reaction on vitelline glands (Fig. 3v, x, xv, xx, xxv). Rabbit anti-somatic antigen strongly reacts with the parenchymal tissue; no positive reaction was found on other organs (Fig. 3xxi–xxv). The scoring of reactive intensities in each area of the parasites is shown in Fig. 3B.

### SDS-PAGE and western blot analysis of tegumental extracts revealed reactive protein of the tegument

Given a strong immunoreactivity with the tegument especially the mice sera, we conducted a tegmental protein extraction and western blot analysis. The tegument was extracted by using Tris-HCl, urea and SDS-Triton X buffers. The protein extracts were separated by SDS-PAGE, thereafter, transferred to the membrane for immunoblot detection. Using *O. viverrini*-infected hamster sera, a strong reactive band at about 100 kDa was observed in the Tris-HCl buffer extracts. Four bands at about 50, 65, 75 and 100 kDa were detected in the urea buffer extracts. Similarly, the SDS-Triton X extracts showed only a prominent band at 95 kDa. Probing by *O. viverrini*-infected mice sera, there was no strong band in the Tris-HCl buffer extracts; however, a prominent band of about 65 kDa was observed in both urea and SDS-Triton X buffers. The protein bands on the SDS-PAGE gel with the size corresponding to these immunoreactive bands were excised for peptide identification by mass spectrometry. The band at 100 kDa of Tris and urea buffers that reacted with hamster sera was identified as a combination of myosin head (OON15278.1) and M60-like mucinase (OON17216). The band at 50 kDa was ATP synthase (OON16694), while the band at 75 kDa was a calpain (XP_009170863). The 65 kDa band in the urea extract that reacts with both hamster and mice sera was identified as SDH (OON19700). Similarly, the 65 kDa band in SDS-Triton X that react with mice sera composed of SDH (OON19700) and innexin (GAA54660).

### Recombinant protein produced in *Escherichia coli* react with *O. viverrini*-infected sera

**Figure 4.**
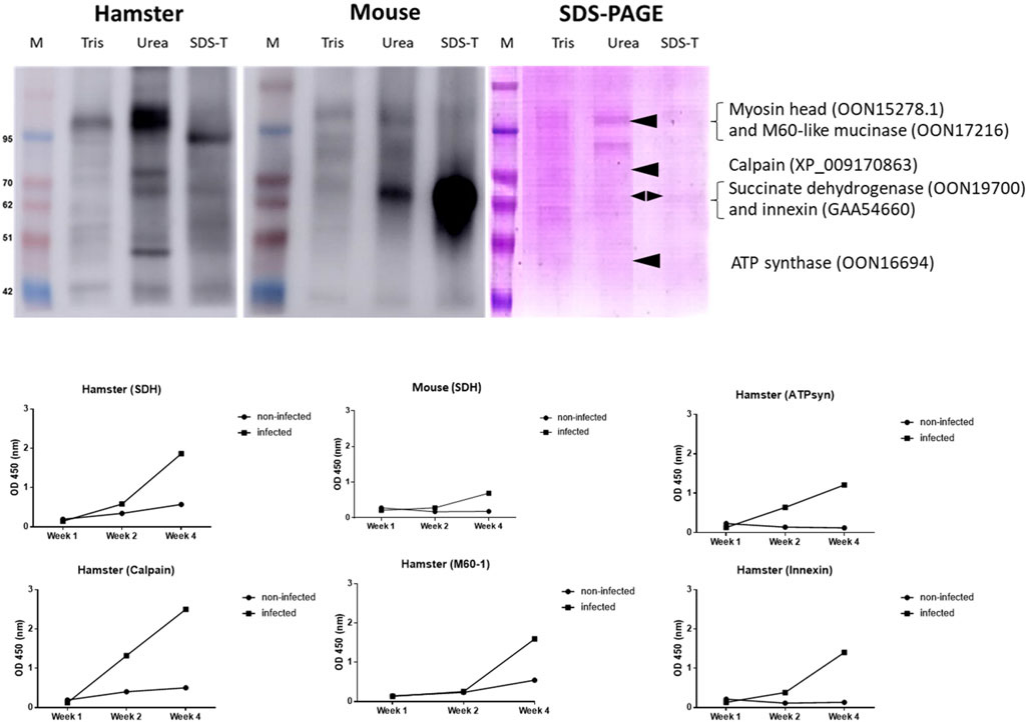
(Top) SDS-PAGE and western blot analysis of tegumental proteins. (Left) Western blot analysis of tegumental protein extracts probed by pooled 4-week *O. viverrini*-infected hamster sera. (Middle) Western blot analysis of tegumental protein extracts probed by pooled 4-week *O. viverrini*-infected mice sera. (Right) Coomassie blue-stained SDS-PAGE gel of tegumental protein extracts and list of proteins [myosin head (OON15278.1), M60-like mucinase (OON17216), ATP synthase (OON16694), calpain (XP_009170863), SDH (OON19700), innexin (GAA54660)] in the band corresponding to the reactive protein of the western blot that have been identified by mass spectrometry. Arrowhead indicates bands corresponding to the reactive bands in the western blot. (Bottom) The ELISA result of the recombinant proteins reacted with the sera of infected mice and hamsters. M, molecular weight markers; Tris, Tris buffer; Urea, urea buffer; SDS-T, SDS-Triton-X buffer; SDH, succinate dehydrogenase; M60-1, M60-like mucinase; ATPsyn, ATP synthase.

To confirm the results from western blot and mass spectrometry, we produced recombinant protein of some of the candidate immunoreactive proteins (M60-like mucinase, calpain, innexin, SDH and ATP synthase) in *E. coli* expression system and tested their immunoreactivity with sera from mice and hamsters by ELISA. The results showed that all recombinant proteins reacted with infected hamsters (Fig. 4). Only SDH reacted with mouse sera (Fig. 4); other proteins were not reactive (data not shown).

## Discussion

We have investigated the immune response of susceptible and non-susceptible hosts against *O. viverrini* proteins. The antibody responses of infected and non-infected hamsters and mice were analysed by ELISA. The results showed that antibodies in mice develop earlier and stronger than those in hamsters. Western blot analysis of the somatic extract revealed that hamster serum showed minimal reactivity with proteins at 70–100 kDa, while mice reacted strongly with 100 kDa band and the higher molecular weight proteins. The IHC demonstrated that hamster antibody reacted strongly with the antigen in the gut of the worm. In contrast, the antibody from mice reacted strongly with the tegument of the worm. These results prompt us to investigate the tegumental protein of the worm. While hamster reacted with several tegumental proteins, mice reacted strongly with a single band. The results from these experiments suggest that there is a different pattern of antibody response in susceptible and non-susceptible hosts.

The antibody response against adult *O. viverrini* hamsters was similar to results from previous studies (Sirisinha *et al*., 1983; Sripa and Kaewkes, 2000*b*). The antibody was detected after 2 weeks. There is no study of antibody response to *O. viverrini* infection in the mouse model. This study showed that mouse antibody rises 1–2 weeks after infection, which is 1 week earlier than for the hamster. A delayed response in the hamster, when compared to the mouse, might indicate immunosuppression, which has been shown in a previous study (Wongratanacheewin *et al*., 1987).

Whether or not antibodies make the mouse less permissive than a hamster is still unknown. Previous studies demonstrated that serum from experimentally infected hamsters could kill both adult and juvenile worms of *O. viverrini* (Flavell, 1981). The author suggested that the serum contains a component(s), possibly a specific immunoglobulin(s) capable of reacting with adult and juvenile parasitic antigens.

We hypothesize that the earlier response of mice may enhance a rapid expulsion of the juvenile worm. The high antibody respond at 2 weeks post-infection might be able to kill a young juvenile parasite. In contrast, a delayed immune response in hamster may allow the worm to develop into adult stage, causing transitions with their protein expressions; hence, the antibody that interacts specifically to the proteins of juvenile worm might not be able to bind to the adult worm, causing the resistant to antibody.

Immunolocalization revealed that the gut and tegument are the 2 main targets of antibodies from mice and hamsters. There is no evidence regarding the main effective antibody target on the worm. However, Sirisinha *et al*. (1986) demonstrated that the serum from experimentally infected hamsters could cause tegument damage and parasite killing *in vitro*, even though tegument shedding could potentially avoid the effects of tegumental target antibodies. To date, there is no report of antibodies binding to the gut of the worm. Nevertheless, it is not impossible for *O. viverrini* grazing on mucosa to ingest the antibody. Indeed, both IgG and IgA were found in the bile of *O. viverrini-*infected hamster. Having said that, whether or not the antibodies could survive the digestive enzyme in the gut is questionable.

The results from this study suggest 2 strategies to develop the vaccine against *O. viverrini*. First, vaccination with immunogenic antigens that have been found to be reactive with hamster serum. A delayed response in native infection may reduce the efficacy of the produced antibody. However, if one can immunize the hamster to acquire the memory to be able to respond to the infection earlier, the hamster might be able to expel the worm in the case of the mice. Second, we can use the same strategy as in mice to immunize the hamster with SDH, which is one of the abundant protein of *O. viverrini* tegument (Mulvenna *et al*., 2010*b*) and highly immunoreactive in this study. Parasites utilize their tegument for essential functional interactions with the host, such as nutrient uptake and environmental sensing (Skelly and Alan Wilson, 2006). The tegument is also the primary site where the parasite defends itself against immune recognition. Targeting the tegumental protein by the mice is a clever strategy to defense against *O. viverrini* infection. Indeed, the host-interactive surface is the target of some successful metazoan parasite vaccines including *O. viverrini* (Knox, 2000; Merrifield *et al*., 2016; Phumrattanaprapin *et al*., 2021).

Apart from the vaccine aspect, the immunoreactive tegumental proteins identified in this study have a high potential to be developed as diagnostic markers for opisthorchiasis. Most of them were also found in tegument extracts or excretory–secretory (ES) products of *O. viverrini* in the previous study (Mulvenna *et al*., 2010*a*). The recombinant proteins of these antigens have 2 advantages over the native somatic and ES products. First, a single antigen can provide more specific detection. Second, it can be produced as needed at low cost.

There were differences in response to the native and recombinant proteins in mice and hamster sera. The overall higher response of the hamster's serum to recombinant proteins, when compared with the native proteins, might reflect the lower amount of the target protein compared with other proteins in the pool of somatic antigens. In contrast, the recombinant protein is the majority of the target protein. The lower response to recombinant SDH in mice may be due to the importance of protein modifications such as glycosylation or lipid residues in antibody recognition or binding.

This study focused on model organisms, which may not represent the human context. Does human serum recognize these targets? Do people with recent primary and chronic infections present with the same antibody profiles? These questions need to be validated and explored in the human context.

In conclusion, there is a different antibody response against *O. viverrini* infection in susceptible and non-susceptible hosts. Further investigation of innate and adaptive immune responses in susceptible and non-susceptible hosts will help elucidate the underlying mechanism of resistance, which will lead to better understand host–parasite interaction and to provide better methods to control opisthorchiasis. In addition, further studies on the human will allow us to learn more about the human response and the potential to be applied in the clinical setting.

## Data Availability

The data that support the findings of this study are available from the corresponding author, SS, upon reasonable request.
